# Transcutaneous vagus nerve stimulation: a bibliometric study on current research hotspots and status

**DOI:** 10.3389/fnins.2024.1406135

**Published:** 2024-08-16

**Authors:** Shiyu Fan, Long Yan, Junfeng Zhang, Yujia Sun, Yulin Qian, Meng Wang, Tao Yu

**Affiliations:** ^1^The First Affiliated Hospital of Tianjin University of Traditional Chinese Medicine/National Clinical Medical Research Center of Acupuncture, Tianjin, China; ^2^Tianjin University of Traditional Chinese Medicine, Tianjin, China

**Keywords:** bibliometrics, transcutaneous vagal nerve stimulation, non-invasive neuromodulation, current state of research, hot trends, visual analysis, Citespace

## Abstract

**Background:**

Transcutaneous Vagal Nerve Stimulation (tVNS) has been used as a promising noninvasive neuromodulation technique for the treatment of various systems.The aim of this study was to analyze the research hotspots and future directions of tVNS in the 21st century by using bibliometric methods.

**Methods:**

The study object was the literature related to tVNS from the Web of Science database from 2000 to May 2024. In order to measure and analyze the number of literature issuance, institutions, authors, countries, keywords, co-citations, and journals of publication, we used VOSviewer, Citespace, Bibliometrix R-package, and Scimago Graphica software. A narrative review of the current research content of tVNS was conducted to gain a better understanding of the current state of the field.

**Results:**

A total of 569 papers were included in the study. The results show that from 2000 to 2024, the number of publications shows an increasing trend year by year, involving a total of 326 research institutions. The United States, China, and Germany are the major research centers. The study identified 399 keywords, which roughly formed 11 natural clusters, revealing that the current hotspots of related research are mainly reflected in 3 areas: intervention efficacy on nervous system diseases, mechanism of action of tVNS, and stimulation mode of tVNS. The top 10 most cited references focus on research into the mechanism of action of tVNS.

**Conclusion:**

The efficacy and safety of tVNS have been confirmed in previous studies, but a standardized tVNS treatment protocol has not yet been developed, and most clinical studies have small sample sizes and lack multicenter and multidisciplinary collaboration. Currently, tVNS is used in the treatment of neurological diseases, psychiatric diseases, cardiovascular diseases, and some autoimmune diseases. It is expected that future research in this field will continue to focus on the application of tVNS in central nervous system diseases and the exploration of related mechanisms, and at the same time, with the rise of non-invasive neuromodulation technology, the application of tVNS in other diseases also has great potential for development.

## 1 Introduction

The Vagus nerve is the 10th pair of cerebral nerves, originating from the nucleus of suspicion and the dorsal nucleus of the vagus nerve, which is widely distributed in the pharynx, ear, and internal organs. The vagus nerve fibers are composed of 20% efferent fibers and 80% afferent fibers, which can transmit sensory information to the central nervous system. It is the longest and most widely distributed cerebral nerve in the human body and belongs to the category of parasympathetic nervous system. It exerts a significant regulatory influence on the human body’s nervous system, respiratory system, circulatory system, and digestive system (Skandalakis et al., 1993; Berthoud and Neuhuber, 2000). As early as the 19th century, American neurologist James Corning first proposed the application of vagus nerve stimulation (VNS) to treat epilepsy (Lanska, 2002). Subsequently, based on VNS, further attempts were made to use Transcutaneous Vagal Nerve Stimulation (tVNS) to intervene in epilepsy, but the above therapies were not popularized due to the lack of a complete theoretical framework and controversy throughout the therapeutic efficacy. Since the 20th century, with the improvement of relevant basic research and revision of the conceptual framework, VNS and tVNS therapies have re-entered the public’s view, and become one of the important neuromodulation therapies in the field of neuroscience (Ventureyra, 2000; Lanska, 2002).

tVNS is a non-invasive neuromodulation technology that affects relevant pathways of the central nervous system through non-invasive stimulation of the vagus nerve branches distributed in the skin sensory zone. It exerts anti-inflammatory, anti-oxidative stress, modulation of cortical excitability and neuroplasticity, regulation of endocrine and the Brain-Gut-Microbiome Axis (BGMA; Colzato and Beste, 2020; Bonaz, 2023; Kumaria and Ashkan, 2023). In addition tVNS may play a role in regulating circulatory control, maintaining arterial pressure, and mediating the vasodilatory component of the cardiovascular reflexes through the oxygen-conserving reflexes, which ultimately serve as neuroprotection. This is related to its involvement in the trigeminal cardiac reflex (TCR; Schaller et al., 2009; Nöthen et al., 2010). Previous studies have demonstrated that tVNS can effectively improve neuropsychiatric disorders, such as headache, epilepsy, stroke, and depression, to some extent (Schindler and Burish, 2022). It can also be used to treat circulatory disorders, such as heart failure and atrial fibrillation (Geng et al., 2022), and digestive disorders, such as inflammatory bowel disease (Gu and Li, 2020). The efficacy and safety of this treatment have been well documented. Therefore it is worthwhile to promote its clinical application.

Bibliometrics employs mathematical and statistical techniques to qualitatively and quantitatively analyze literature characteristics, revealing knowledge structures, research trends, and hotspots in a given field (Luo, 1994). VOSviewer, Citespace, Bibliometrix R-package, and Scimago Graphica have powerful visualization and data analysis capabilities and are now widely used in bibliometric analysis (Zeng et al., 2023). This study employs bibliometrics to analyze collaborative network analysis and keyword analysis of relevant literature on tVNS in the 21st century. We present an overview of the current state of research in this field, identify key research topics, and suggest future research directions (Broadly, this includes mechanistic studies of tVNS, standardization of stimulation parameters and efficacy in neurological disorders.). This analysis is a reference for subsequent research endeavors and the formulation of relevant diagnosis and treatment plans in this field.

## 2 Materials and methods

### 2.1 Search strategy and data collection

A computerized search was conducted on Web of Science for literature pertaining to tVNS from 2000 to May 25, 2024, using the English search strategy outlined in Supplementary Table 1. The search was limited to the ‘Web of Science Core Collection,’ resulting in the retrieval of 914 English articles. These articles were manually screened based on predefined inclusion and exclusion criteria, with 569 articles meeting the inclusion criteria.

#### 2.1.1 Inclusion criteria

·Publicly available journal literature related to tVNS (excluding invasive vagus nerve stimulation), with the type of “Article” and a few high relevance ‘Review’. The studies included in this article for analysis were selected from the literature on the basis of their relevance to the topic under discussion. They comprise clinical trials, animal experiments, and case studies. The objective is to elucidate the clinical efficacy or mechanism of action associated with taVNS.

#### 2.1.2 Exclusion criteria

- Excluded duplicates and literature lacking complete information or relevance to the topic.
- Excluded document types such as conference papers, books, book chapters, newspapers, news, and conference reviews.
- No restrictions were imposed based on geography or language; however, documents with incomplete information and duplicate publications were excluded.

### 2.2 Data processing flow and parameter setting

The scientometric analysis in this study utilized various tools: CiteSpace software (version 6.1.6), VOSviewer (version 1.6.19), Bibliometrix R-package, and Scimago Graphica (version 1.0.38). Literature from Web of Science was exported in Refworks format, processed through CiteSpace software, and converted to _xxx.txt format for compatibility. The following steps were conducted:

1. CiteSpace 6.1.6 was used for de-duplication, resulting in 569 documents.
2. Manual data merging resolved discrepancies in country names and author variations.
3. Microsoft Excel 2013 facilitated the mapping annual publication trends and the categorization of keywords, authors, institutions, and countries.
4. CiteSpace 6.1.6 was used for data conversion and analysis, with parameters set for a time frame from 2002 to 2024, and specific criteria for node selection and threshold settings. Keyword emergence graphs were plotted with Burstness- γ (0.8) for comprehensive mapping.
5. VOSviewer 1.6.19 was employed for author collaboration network visualization, using Refworks text format with a co-occurrence minimum frequency of 5.
6. Scimago Graphica 1.0.38 was utilized to analyze and visualize publication distribution across different regions and countries.
7. Bibliometrix R-package was applied to summarize cited literature and journal distribution information.

## 3 Results

### 3.1 Analysis of trends in publications

**Figure 1:**
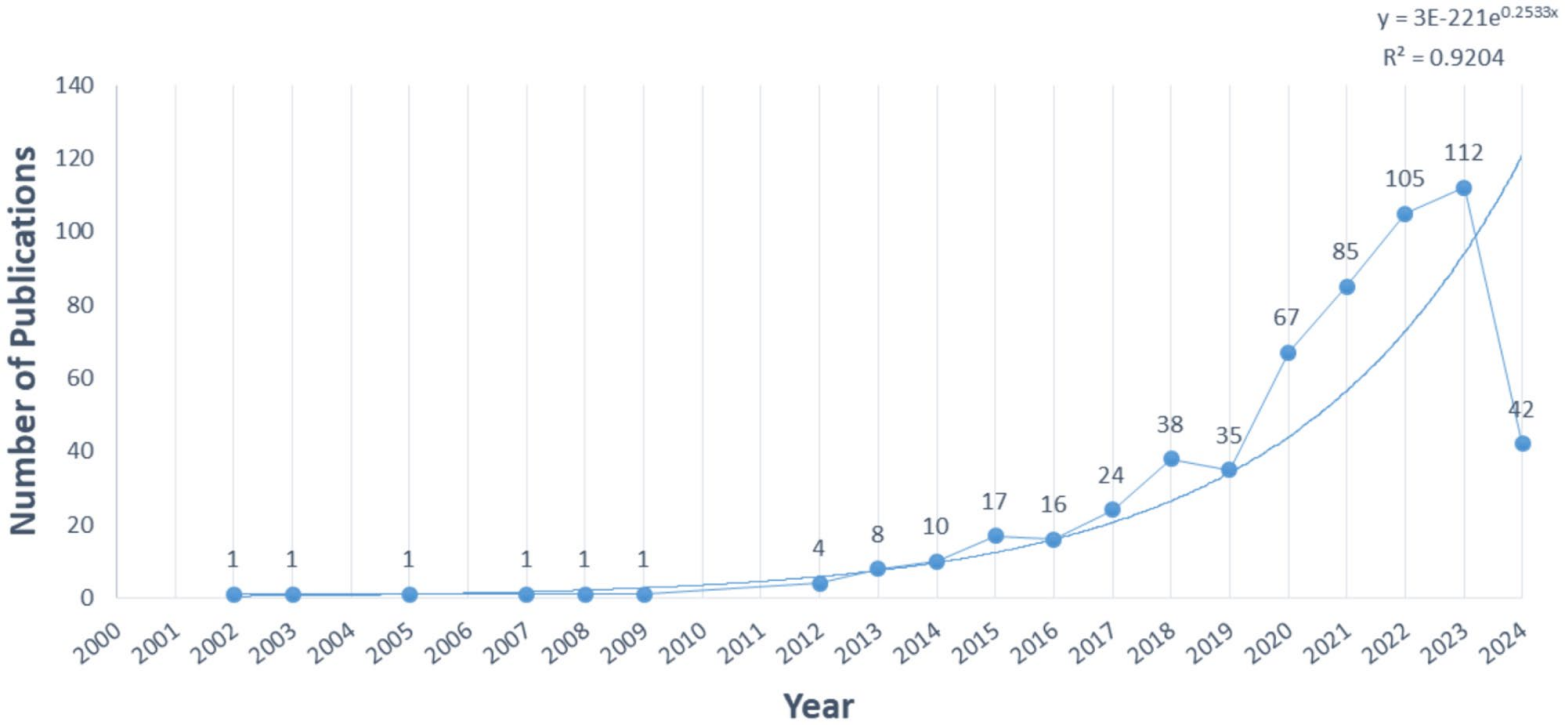
The trend of the annual published articles of tVNS from 2000 to 2024.

The annual publications (Figure 1) serve as an indicator of the field’s development. tVNS-related research has exhibited progressive growth since the 21st century. From 2000 to 2012, the average annual publications were low (1/year), indicating an embryonic stage. Between 2012 and 2019, a slow but steady increase occurred, averaging 19 publications/year. Since 2019, the average annual number of publications has grown rapidly. The 5-year growth rate reaches 33.7% and peaks in 2023 (112 articles). Exponential regression analysis reveals a positive correlation (y = 3E-221e0.2533x, R2 = 0.9204), suggesting sustained research interest in the future.

### 3.2 Author collaboration analysis

**Figure 2:**
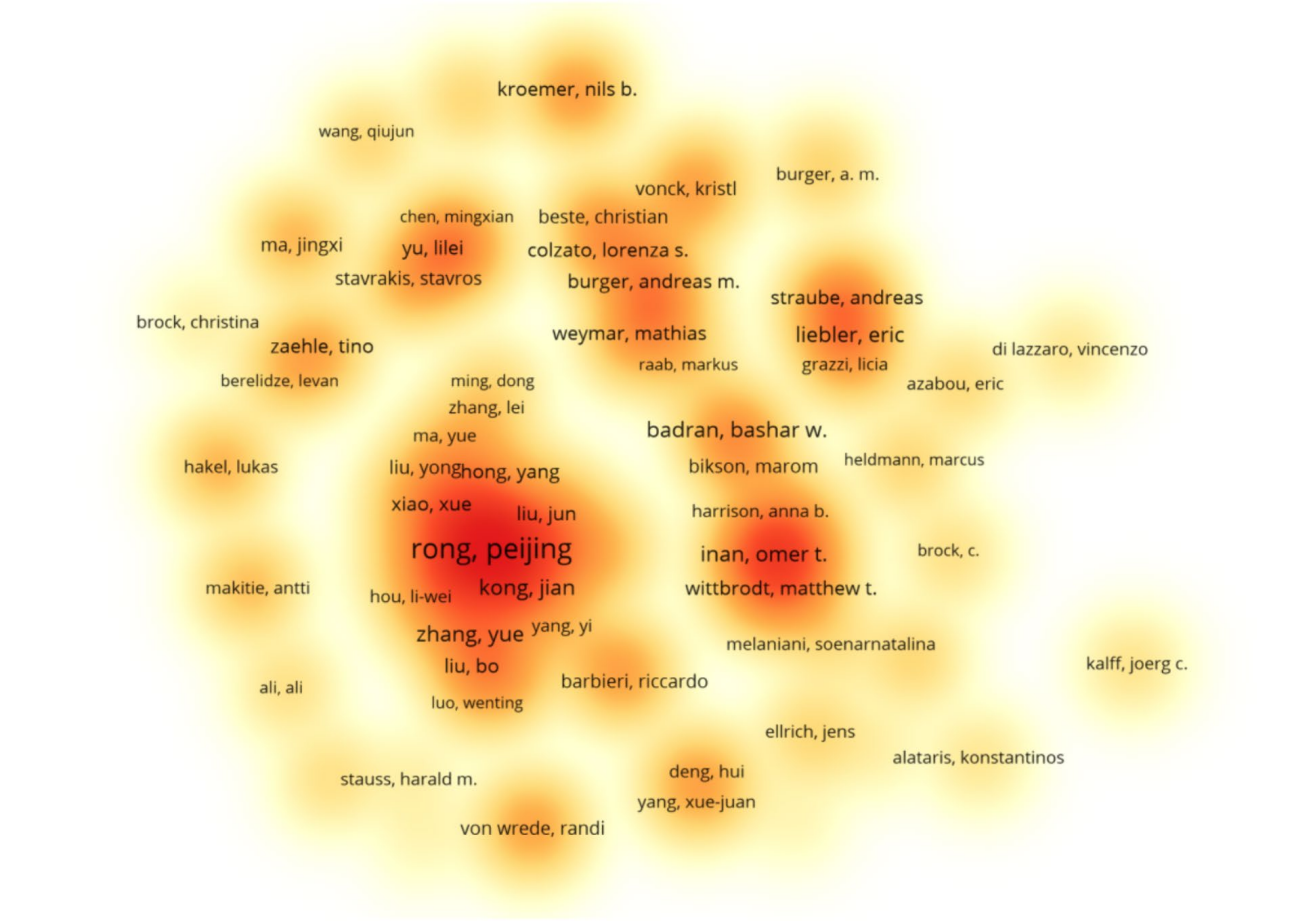
Occurrence of authors. The closer the color of the area is to red, the higher the density of authors in the area, and the scale denoting the number of documents.

To ensure data analysis accuracy, we examined raw data using Microsoft Excel 2013. Name variants for the same author were manually merged for precision. Results revealed 569 publications involving 2,655 authors in this study. The top contributors were from the Department of Functional Research, Institute of Acupuncture and Moxibustion, China Academy of Chinese Medical Sciences, particularly Peijing Rong, with 52 publications. Supplementary Table 2 displays the top 10 authors and their affiliations. Following Price’s law, authors with more than 5 publications are considered core contributors (Ninkov et al., 2022). According to the search results, there are 106 core authors in this study. Utilizing VOSviewer software for author density mapping (Figure 2), a network of author collaborations with 106 nodes was generated. After removing isolated points, 18 clusters emerged, suggesting a network of authors led by Peijing Rong, Liebler Eric, Badran Bashar W, Burger Andreas, and others, forming the research team.

### 3.3 Analysis of institutional cooperation

**Figure 3:**
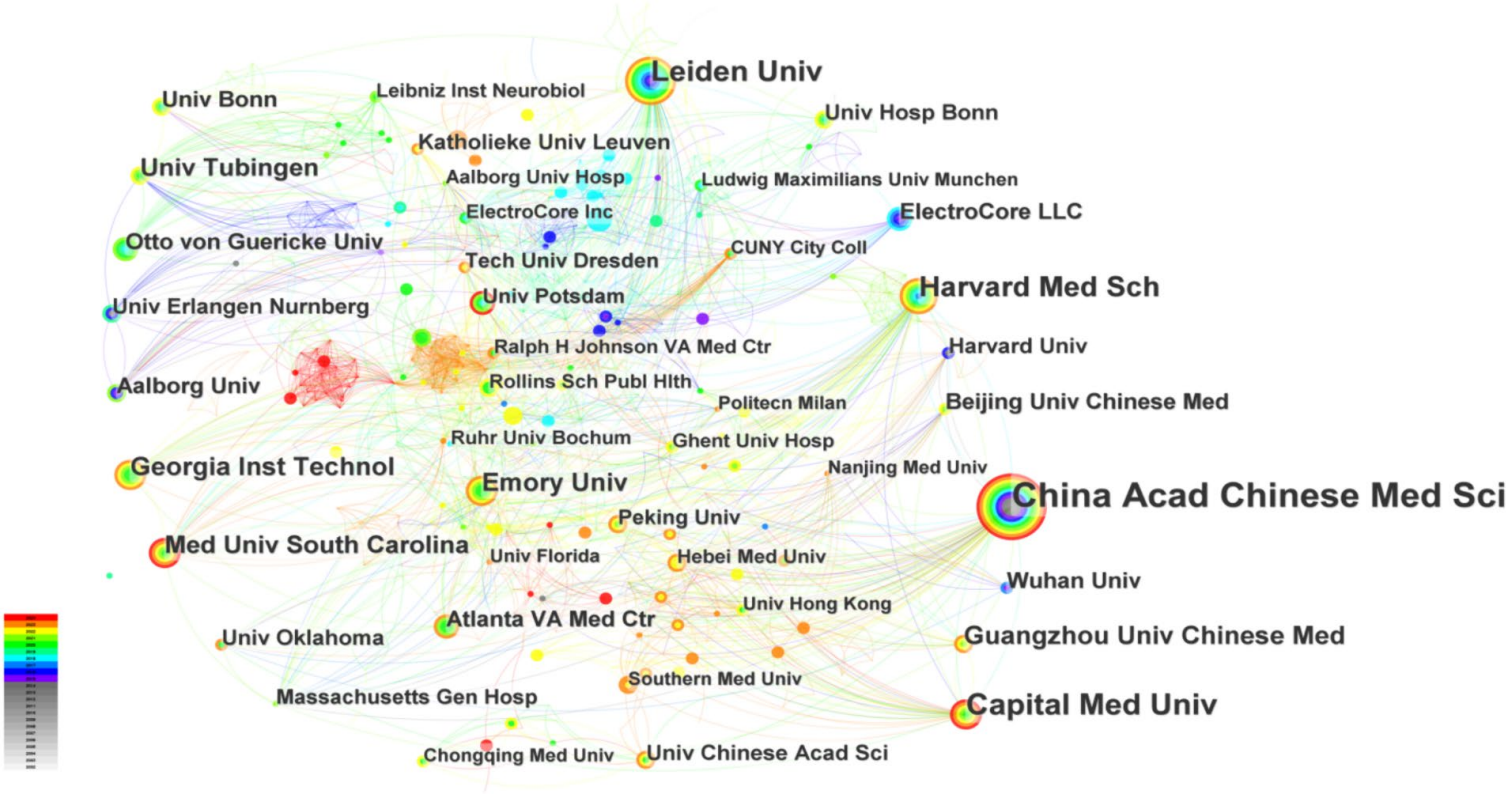
Occurrence of institutions. The thickness of the line indicating collaboration strength, and the circle size denoting the number of articles.

Citespace is used to analyze the collaborating institutions. This study encompasses 326 institutions cited in the literature (Figure 3), resulting in 682 connections among them. The network density of institutional collaboration stands at 0.0126, indicating a relatively dispersed collaboration among institutions. It is evident that research institutions, primarily in the medical schools, predominate, forming collaborative groups centered around institutions such as China Acad Chinese Med Sci, Harvard Med Sch, Leiden Univ, and Med Univ South Carolina, with China Acad Chinese Med Sci leading in publication output (60 papers). Due to geographical and academic factors, collaboration between Chinese institutions and those in Europe and America remains relatively limited. A tightly woven mesh of collaborative relationships has yet to emerge.

### 3.4 Analysis of country cooperation

**Figure 4:**
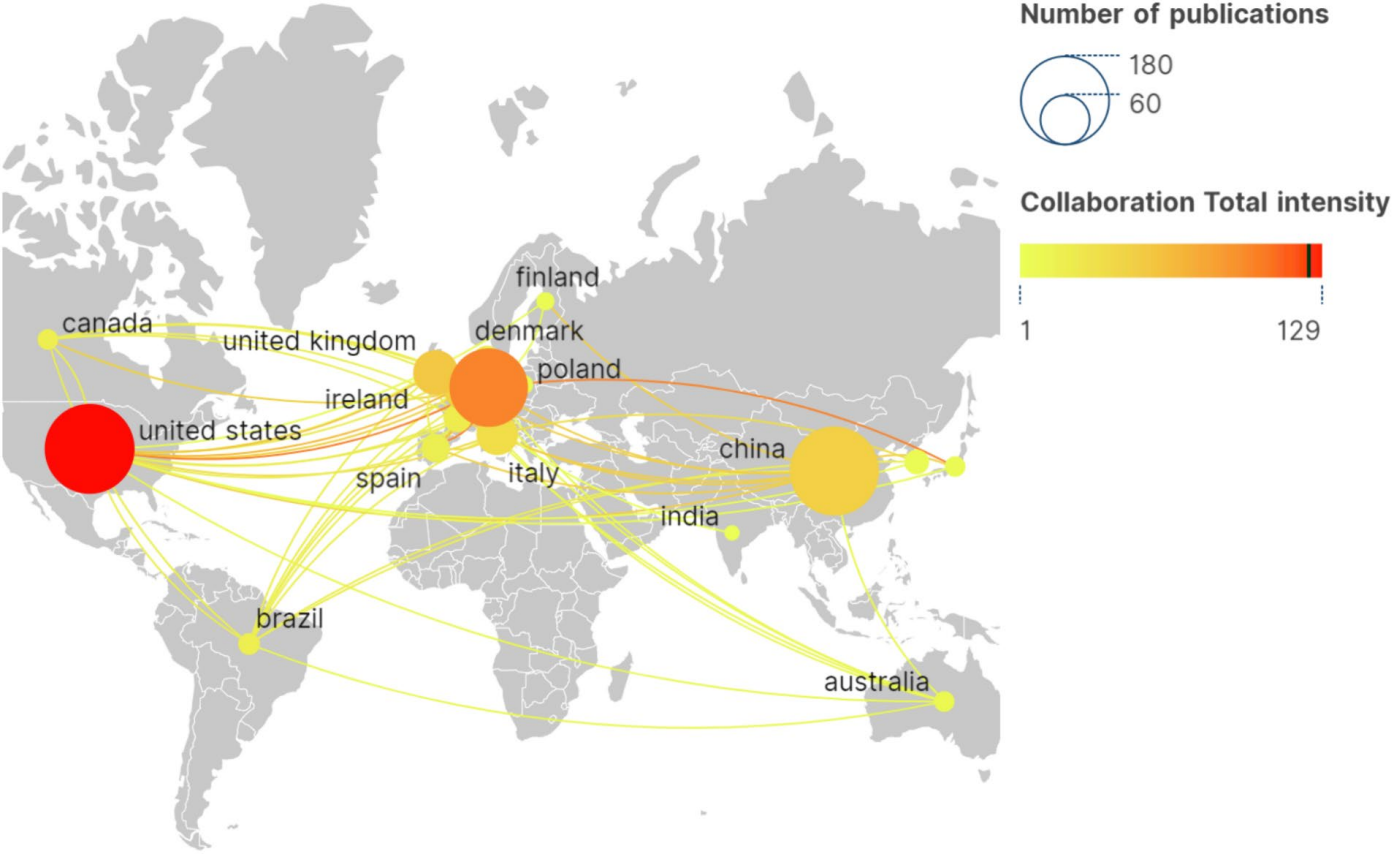
International collaborative network for geovisualization. The circle color indicating collaboration strength, and the circle size denoting the number of articles.

Scimago Graphica is used to make a national collaboration analysis. This study engaged researchers from 52 countries. Table 1 highlights the top 10 countries by publication count, led by the United States (*n* = 172), followed by China (*n* = 169) and Germany (*n* = 131). The U.S. holds the highest total citations at 5115. In Figure 4, the collaboration network among countries/regions is depicted, with circle color indicating collaboration strength, and the circle size denoting the number of articles. According to a comprehensive analysis, the United States is recognized as a significant contributor to international cooperation, with the highest number of articles issued and the total number of citations. Furthermore, the United States has established a close cooperation with China, the United Kingdom, Germany, Italy, and other nations.

**Table 1:** The top 10 countries in tVNS research.

Rank	Number of publications/article	Citations	Country
1	172	5,115	United States
2	169	2,894	China
3	131	4,967	Germany
4	43	1,526	United Kingdom
5	39	1,317	Netherlands
6	37	815	Italy
7	33	790	Belgium
8	17	1,363	Denmark
9	17	259	Spain
10	17	209	France

### 3.5 Keyword visualization and analysis

#### 3.5.1 Co-occurrence analysis

**Figure 5:**
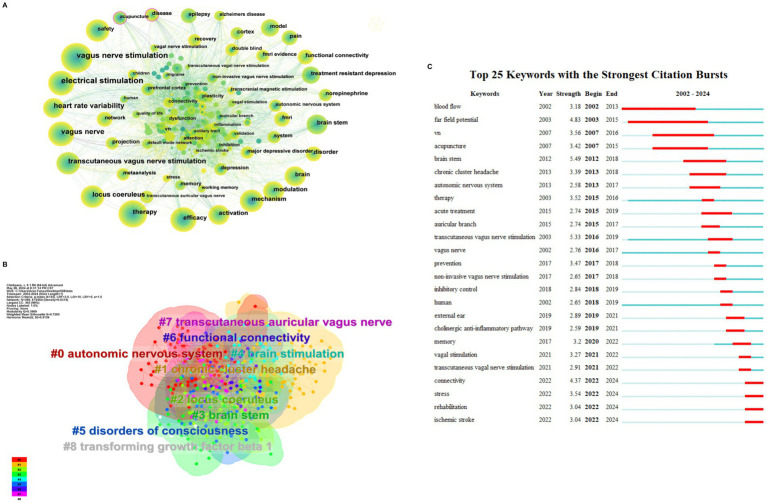
**(A)** Keywords occurrence. The thickness of the line indicating collaboration strength, and the circle size denoting the number of articles. **(B)** Keyword clustering. The color of the region represent cluster. The size of the area represents the number of keywords that are included. **(C)** Top 25 keywords with the strongest citation bursts.

The article’s keywords have been condensed to effectively summarize the core research content. The presence of high-frequency keywords may indicate a hot research direction. As depicted in Figure 5A, the keywords have been visualized and analyzed using Citespace. The total number of keywords is 399, with 2,504 links, and a network density of 0.0315, which suggests a high literature density. Table 2 presents the top 10 keywords, excluding low-information words such as ‘therapy’, 'efficacy’, and ‘vagus nerve’. The keywords can be broadly categorized into the mechanism of action, stimulation mode, therapeutic diseases, and other categories. Currently, the mechanisms of action related to tVNS include activation of the noradrenergic neural pathway originating from the blue spot and the cholinergic anti-inflammatory pathway (CAP), among others (Urbin et al., 2021; Agarwal et al., 2024). The stimulation modes included transcutaneous vagus nerve electrical stimulation, transcutaneous vagus nerve magnetic stimulation and so on (Zhang et al., 2023).

**Table 2:** Keyword frequency and center value in tVNS research.

Rank	Literature volume/article	Centrality	Keywords
1	141	0.12	Electrical stimulation
2	80	0.08	Heart rate variability
3	66	0.07	Locus coeruleus
4	43	0.13	Activation
5	38	0.09	Mechanism
6	38	0.09	Brain stem
7	35	0.02	Disorder
8	32	0.06	Treatment resistant depression
9	29	0.02	Norepinephrine
10	28	0.02	Pain

#### 3.5.2 Cluster analysis

The keyword clustering analysis (Figure 5B) can show a highly condensed keyword clustering plate. To a certain extent, it reflects the knowledge structure and research hotspots in this field, which is conducive to grasping the research direction (Huifeng, 2017). The K-clustering algorithm, utilizing the LLR approach, was employed to cluster the keywords associated with tVNS. A total of 11 clustering results were obtained for the keywords in this study. The clustering module value, Modularity Q = 0.3969 > 0.3, indicates the presence of a significant clustering structure. The silhouette value, Silhouette (S) = 0.7285 > 0.7. This indicates that the clustering results are highly convincing and can reflect the research hotspots in this field to a significant extent (Yue et al., 2015). The overlapping and interlacing between the clusters indicate that the clusters are more closely linked to each other. Table 3 presents the top nine keyword clusters. Clusters #4 and #7 reveals different tVNS stimulation methods, in which transcutaneous auricular vagus nerve stimulation (taVNS) has been the subject of considerable research interest. Clusters #1 and #5 illustrate the advantageous diseases that can be treated with tVNS intervention, with a primary focus on the treatment of neurological disorders. The clusters #0, #2, #3, #6, and #8 demonstrate the mechanism of action of tVNS. This indicates that the regulation of the autonomic nervous system, the functional connectivity between various brain regions, and the CAP pathway associated with the locus coeruleus in the brainstem are the current research focus.

**Table 3:** Keyword clustering in tVNS research.

Cluster ID	Size	Silhouette	Year	Term label
0	73	0.743	2016	Autonomic nervous system
1	72	0.73	2016	Chronic cluster headache
2	57	0.659	2018	Locus coeruleus
3	48	0.683	2016	Brain stem
4	38	0.809	2014	Brain stimulation
5	36	0.673	2019	Disorders of consciousness
6	36	0.713	2018	Functional connectivity
7	27	0.838	2015	Transcutaneous auricular vagus nerve
8	6	0.976	2015	Transforming growth factor beta 1

#### 3.5.3 Keyword with citation bursts analysis

Figure 5C illustrates the emergence of keywords that are used more frequently or suddenly appear during the development of the field. This analysis provides insight into the research process over time and the historical span of the literature. The Citespace software was utilized to identify 25 emergent words in the included literature. Previous research has mainly concentrated on investigating the therapeutic effects of tVNS, which involves assessing its effectiveness through measures such as “blood flow” and vagus nerve sensory evoked potentials. After 2010, the research direction of the field gradually shifted toward clinical trials of tVNS for different diseases, including cluster headache, stroke. In recent years, there has been an increase in studies on the stimulation mode and mechanism of action related to tVNS. Furthermore, the exploration of the anti-inflammatory mechanism in tVNS has become a focus of research and future research in this area will be more intensive.

### 3.6 Analysis of co-cited literature

**Figure 6:**
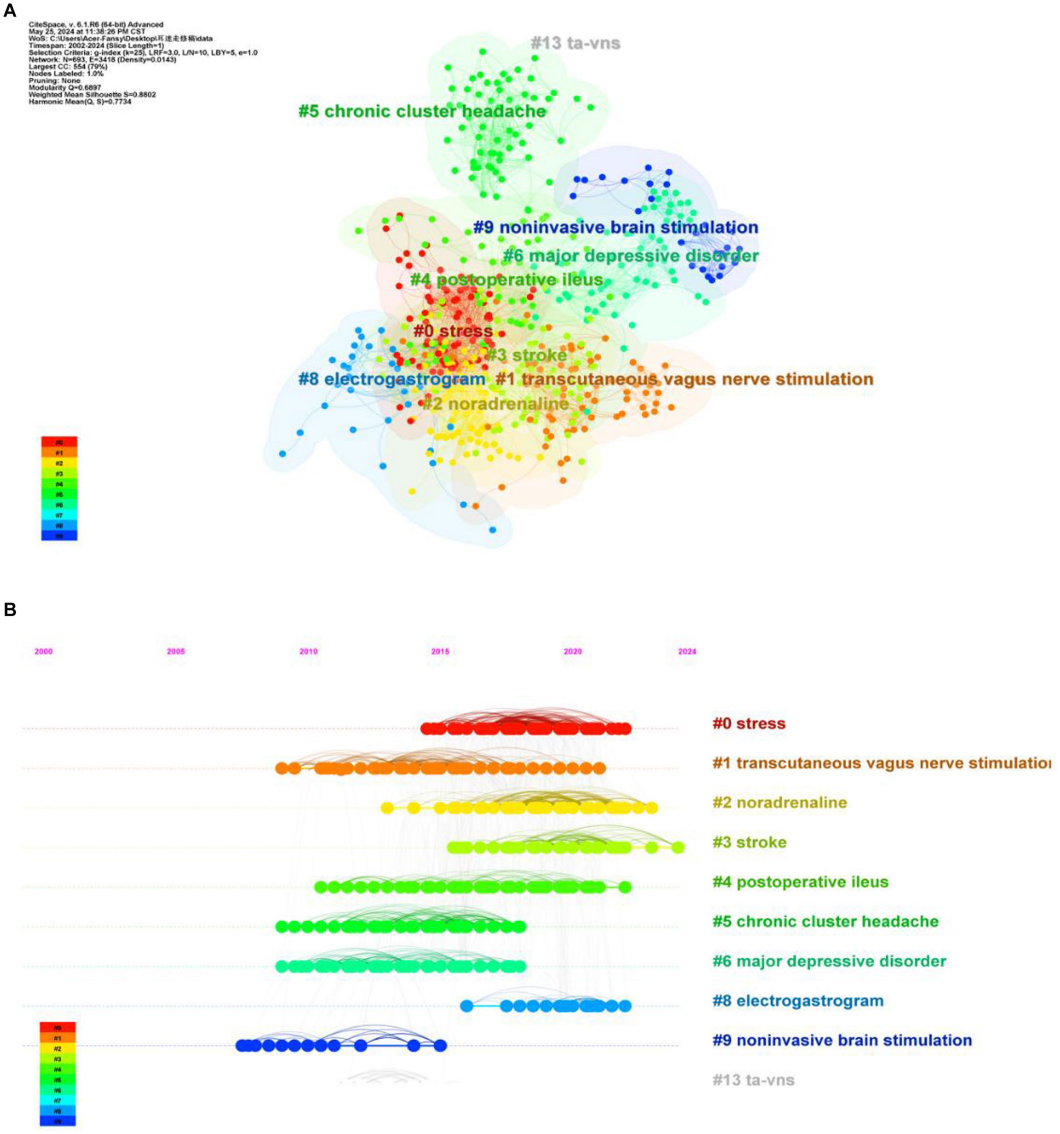
**(A)** Clustering map of cited literature. The color of the region represent cluster. The size of the area represents the number of keywords that are included. **(B)** Timeline chart of cited literature.

The analysis of the characteristics of the co-cited literature is beneficial in understanding the current research hotspots and frontiers in this field, as well as exploring future research directions (Zhilong et al., 2023). The Bibliometrix R-package was employed to analyze the number of citations of related literature published in this field between 2000 and 2024. Table 4 presents the 10 most highly cited articles, all of which have been cited more than 100 times. This indicates that these articles have high academic and reference values and are widely recognized. “Non-invasive Access to the Vagus Nerve Central Projections via Electrical Stimulation of the External Ear: fMRI Evidence in Humans,” published by Frangos in 2015, was ranked first (cited 396 times). Employing Citespace for co-cited literature clustering revealed the top 10 clusters (Figure 6A; Q = 0.6897, S = 0.8802), suggesting high clustering confidence. The integration of the clustering outcomes with the timeline (Figure 6B) reveals recent research directions in the fields of stroke (#3), noradrenaline (#2), and stress (#0).

**Table 4:** The top 10 references in tVNS research.

Rank	Author	Year	Title	Total citations	TC per year
1	Frangos E	2015	Non-invasive Access to the Vagus Nerve Central Projections via Electrical Stimulation of the External Ear: fMRI Evidence in Humans	396	39.60
2	Clancy JA	2014	Non-invasive vagus nerve stimulation in healthy humans reduces sympathetic nerve activity	275	25.00
3	Kraus T	2007	BOLD fMRI deactivation of limbic and temporal brain structures and mood enhancing effect by transcutaneous vagus nerve stimulation	274	15.22
4	Yakunina N	2017	Optimization of Transcutaneous Vagus Nerve Stimulation Using Functional MRI	237	29.63
5	Fang JL	2016	Transcutaneous Vagus Nerve Stimulation Modulates Default Mode Network in Major Depressive Disorder	212	23.56
6	Kraus T	2013	CNS BOLD fMRI effects of sham-controlled transcutaneous electrical nerve stimulation in the left outer auditory canal - a pilot study	196	16.33
7	Dietrich S	2008	A novel transcutaneous vagus nerve stimulation leads to brainstem and cerebral activations measured by functional MRI	185	10.88
8	Stefan H	2012	Transcutaneous vagus nerve stimulation (t-VNS) in pharmacoresistant epilepsies: a proof of concept trial	171	13.15
9	Silberstein SD	2016	Non-Invasive Vagus Nerve Stimulation for the ACute Treatment of Cluster Headache: Findings From the Randomized, Double-Blind, Sham-Controlled ACT1 Study	171	19.00
10	Straube A	2015	Treatment of chronic migraine with transcutaneous stimulation of the auricular branch of the vagal nerve (auricular t-VNS): a randomized, monocentric clinical trial	168	16.80

### 3.7 Analysis of journals

According to the statistical analysis conducted using the Bibliometrix R-package, Table 5 presents the top 10 journals with the highest number of publications in the field of tVNS. Among these journals, ‘BRAIN STIMULATION’ (IF = 7.70, Q1) stands out with 44 papers related to tVNS. The article has received a considerable number of citations, totaling 2,441, which highlights its significant influence in this research domain.

**Table 5:** The top 10 journals in tVNS research.

Rank	Journals	Documents	h_index	Total citation	IF, JCR [2022]
1	Brain Stimulation	44	22	2,441	7.7,Q1
2	Frontiers in Neuroscience	28	9	249	4.3,Q3
3	Scientific Reports	18	9	226	4.6,Q2
4	Frontiers in Neurology	16	6	143	3.4,Q3
5	Autonomic Neuroscience-Basic & Clinical	14	8	196	2.7,Q4
6	Neuromodulation	14	8	392	2.8,Q3
7	Frontiers in Human Neuroscience	14	6	195	2.9,Q3
8	Plos one	10	8	220	3.72,Q2
9	Brain Sciences	10	6	74	3.3,Q3
10	Journal of Headache and Pain	9	9	493	7.4,Q1

**Figure 7:**
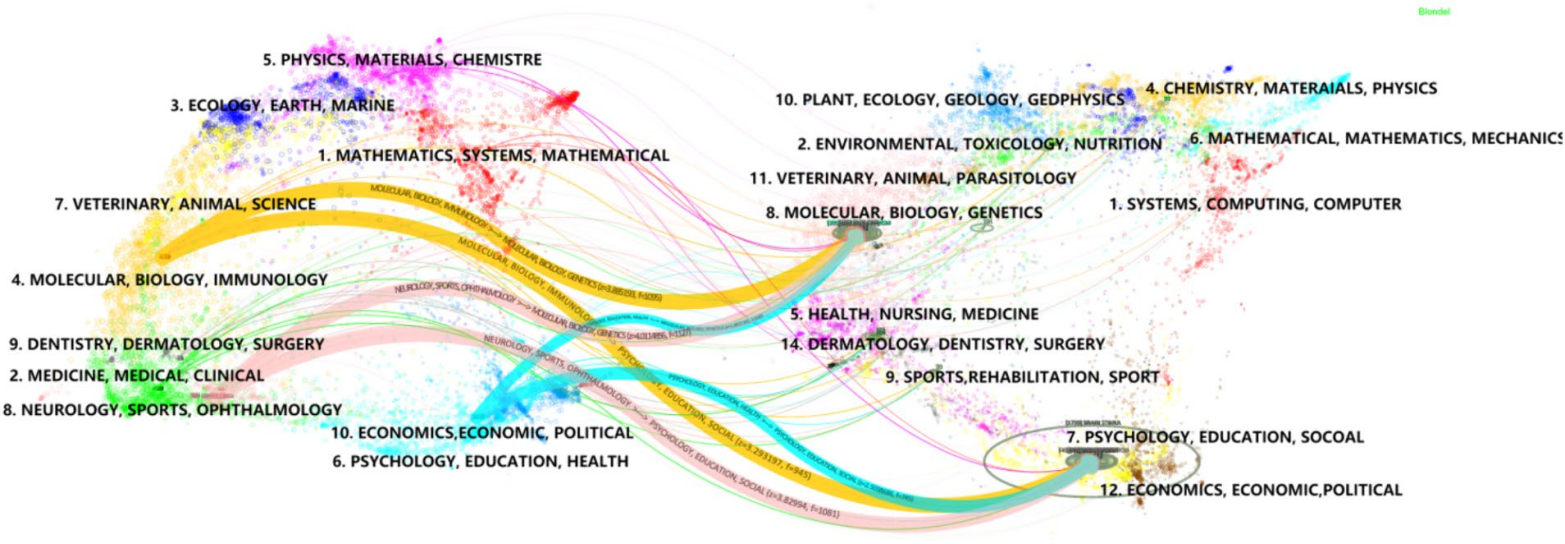
A dual-map overlay of the journals on tVNS research. The map of cited journals is superimposed on top of the map of citing journals, with the former being the iterative map and the latter being the base map. The labels represent the disciplines covered by the journals, and the colored paths represent citation relationships.

Using Citespace software for dual-map overlay journal network analysis, the dual-map overlay function demonstrates the knowledge flow between citing and cited journals (Figure 7), revealing a scientific hybrid model of the global journal map in this field (Wei et al., 2022). After Z-score correction, the results indicate that MOLECULAR/BIOLOGY/GENETICS/PSYCHOLOGY/EDUCATION/SOCIOLOGY serves as the primary citation pathways in cited journals. It forms the main theoretical and technical basis for research with limited interdisciplinary studies and the strongest correlations (Chen et al., 2024). Among citing journals, NEUROLOGY/SPORTS/OPHTHALMOLOGY represent the top three citation pathways, indicating high citation frequency and relevance in these disciplines’ research. When these disciplines are used as source journals, molecular biology, biology, and genetics are the most frequently cited clusters with a Z-score of 4.00. This suggests a strong interconnection and high citation rates between these fields.

## 4 Discussion

In this study, we visualize and analyze the number of publications, authors, countries, institutions, references, and published journals of tVNS-related literature. Furthermore, we conducted a comprehensive and systematic review of the latest research directions based on the findings of the visualization analysis, with the aim of providing a detailed account of the advancements made in the field of tVNS.

### 4.1 Analysis of the current status of tVNS research

According to the data, the annual publication volume has been steadily increasing since the 21st century, with a peak in 2021–2023. It is reasonable to assume that there will be continued potential for development in this area in the future. Upon analyzing the number of author publications and their collaboration network, it appears that Peijing Rong and her team have published a significant number of works in this field. A recent study has investigated the effects of taVNS on gastric motility injury and autonomic mechanisms (Zhu et al., 2023). Furthermore, they have investigated the regulatory effects of taVNS on brain networks and neural activities in patients with mild, moderate, and severe depression (Ma et al., 2023). It is worth noting that the United States and China had the highest number of publications. The United States plays a leading role in tVNS research, as demonstrated by its extensive academic collaborations with other nations. From an institutional perspective, three of the top 10 institutions are located in China, which is consistent with the distribution of publications by country. However, cross-regional collaboration is limited and may hinder global research exchange and integration. Highly cited publications often indicate the research field’s theme (Jiang et al., 2023). This study provides a summary of the 10 most frequently cited publications since the 21st century, which mainly focus on the mechanism of action and clinical application of tVNS. The article published in BRAIN STIMULATION has been cited most frequently. It demonstrates that the central projection of the ear branch of the vagus nerve is consistent with the classical central vagal projection and can act in a non-invasive manner through the outer ear (Frangos et al., 2015). According to the distribution of journals, BRAIN STIMULATION and FRONTIERS IN NEUROSCIENCE have the highest number of articles and citations in this field. This indicates that there is significant potential for further development of tVNS research in neuroscience.

### 4.2 Content analysis of tVNS research

The objective of this study is to examine the prevalent research areas of tVNS. Keywords are brief summaries of literature content that facilitate in-depth exploration of research hotspots and development trends (Wang et al., 2023). Based on keyword and citation clustering themes, which are divided into three relevant aspects of tVNS research content, including tVNS mechanisms of action, stimulation patterns, and clinical applications. The following is an in-depth discussion of the keywords that appear more frequently in the results of this bibliometric study, as well as the results achieved by representative research teams in the field. Moreover, this study also investigates the safety of tVNS applications.

#### 4.2.1 Mechanism of action of tVNS (keyword clusters #0, #2, #3, #6, #8)

Keyword clusters #0, #2, #3, #6, #8 are relevant with the mechanism of tVNS. The findings of our study indicate that the investigation of anti-inflammatory mechanisms, functional connectivity in brain regions and locus coeruleus-norepinephrine (LC-NE) system represents a current area of interest.

1. Anti-inflammatory mechanism

The vagus nerve is a major component of the neuroendocrine-immune axis, participating in the regulation of neural, behavioral, and endocrine responses. As the frontline of innate defense against infections/inflammations in the body, it helps maintain internal homeostasis (Johnston and Webster, 2009). The anti-inflammatory pathways of tVNS mainly include CAP, the splenic sympathetic anti-inflammatory pathway, and the hypothalamic–pituitary–adrenal (HPA) axis anti-inflammatory pathway (Yan et al., 2024).

① CAP anti-inflammatory mechanism

The result of keyword with citation bursts analysis showed that ‘cholinergic anti-inflammatory pathway’ appears in large numbers during 2019–2021. CAP refers to the excitation of the vagus nerve by external stimulation, leading to the production of the anti-inflammatory neurotransmitter acetylcholine (ACh) at the vagus nerve endings, which activates alpha-7 nicotinic acetylcholine receptors (α7nAChR) on monocytes and macrophages (Hongxian et al., 2023). This further activates the nuclear factor kappa B (NF-κB) signaling pathway and the Janus kinase 2/signal transducers and activators of transcription 3 (JAK2/STAT3) pathway within the cells, suppressing the production of cytokines such as IL-1β and TNF-α, ultimately exerting anti-inflammatory effects (Wang et al., 2003). Previous animal studies have suggested that by enhancing CAP, tVNS can counteract colon cancer induced by 1,2-dimethylhydrazine (DMH). The research results showed that applying tVNS could restore DMH-induced mitochondrial apoptosis. At the protein and mRNA levels, tVNS can activate CAP by upregulating the expression of α7nAChR, downregulating nuclear factor kappa B p65 (NFκBp65) in activated B cells, and enhancing the expression of tissue necrosis factor-alpha and high mobility group box-1 (Rawat et al., 2019). Aranow et al. (2021) conducted a 12-day randomized controlled study and concluded that taVNS significantly improves inflammatory musculoskeletal pain and fatigue in patients with systemic lupus erythematosus. The mechanism of action may involve the activation of the CAP, leading to a reduction in plasma levels of substance P in subjects. This suggests that plasma substance P may be more sensitive to the biological response of taVNS than cytokines, which is a worthy area for further exploration. In an ischemic stroke model, taVNS is believed to modulate brain ischemia/reperfusion (I/R) injury, axonal plasticity, and vascular regeneration, thereby improving neuro-motor function. Its molecular mechanism involves taVNS activating the CAP pathway, thereby activating downstream PPAR-γ (expressed mainly in neurons and astrocytes) nuclear transcription factors to promote the expression and secretion of brain-derived neurotrophic factors (BDNF) and vascular endothelial growth factors (Li, 2020).

② HPA axis

The HPA axis is one of the crucial neuroendocrine axes in the human body. Stimulation of the vagus nerve, on the one hand, can reduce peripheral inflammatory responses by activating the HPA axis (Kaniusas et al., 2019). According to the anatomy of the vagus nerve, peripheral inflammatory factors including interleukin (IL)-1, IL-6, and TNFα can transmit information to the nucleus of the solitary tract (NTS) through vagal afferent fibers, activating neurons located in the A2 noradrenergic group and projecting information to the paraventricular nucleus of the hypothalamus (PVH). This pathway can activate neurons producing corticotropin-releasing factor (CRF) in this area, stimulating the hypothalamus to release corticotropin-releasing hormone via the HPA axis, ultimately leading to adrenal release of glucocorticoids, thereby reducing peripheral inflammation (Bonaz et al., 2016). In terms of anti-inflammatory effects, taVNS shares similar physiological effects with VNS. Studies have shown that taVNS can reduce pro-inflammatory cytokines, modulate lung injury, and alleviate acute respiratory distress syndrome caused by COVID-19 by activating the HPA anti-inflammatory pathway and the CAP (Kaniusas et al., 2020). On the other hand, the HPA axis is the primary stress response system in the human body and is closely associated with the pathophysiology of depression. Long-term stress, including psychological and physiological stress as well as other stimulation, can lead to HPA axis hyperactivity, resulting in abnormal secretion of plasma cortisol (CORT) and adrenocorticotropic hormone (ACTH), thereby inducing depressive-like behavior (Liyanarachchi et al., 2017). Previous animal studies have shown that taVNS can improve depressive states in rats by inhibiting excessive activity of the HPA axis (Hou et al., 2022). Recent clinical research has found that tVNS can upregulate salivary cortisol levels and decrease salivary flow rate in temporal lobe epilepsy patients, improving epilepsy symptoms by suppressing excessive activation of the HPA axis and the autonomic nervous system (Doerr et al., 2023). The study by Li et al. (2020) demonstrated that 20 Hz taVNS is an effective treatment for depressive-like behavior and downregulates the excessive activity of the HPA axis.

③ Splanchnic sympathetic anti-inflammatory pathway

The stimulation of the vagus nerve has been demonstrated to activate splanchnic sympathetic anti-inflammatory pathways and to inhibit the secretion of pro-inflammatory factors in splanchnic macrophages (Kaniusas et al., 2019). Studies have shown that taVNS may inhibit peripheral inflammatory responses, reduce the release of the chemokine CXCL1, and ameliorate lipopolysaccharide (LPS)-induced depressive-like behaviors in rats by activating the splenic α7n Ach R/JAK2/STAT3 signaling pathway (Yu et al., 2023). However, the splanchnic sympathetic anti-inflammatory pathway has been questioned by some researchers. Martelli et al. (2014) demonstrated that plasma levels of the key inflammatory mediator tumor necrosis factor-alpha (TNFα) in rats injected intravenously with lipopolysaccharide were not affected by previous bilateral cervical vagotomy. However, the level of TNFα was increased by about 5-fold after severing visceral sympathetic nerves. This suggested that the inflammatory response after LPS immune attack is not reduced by reflex activation of the splanchnic sympathetic anti-inflammatory pathway by the vagus nerve. Instead, its anti-inflammatory mechanism may be related to splanchnic nerve activity driven by the conventional sympathetic pathway via the visceral nerve. However, the above-mentioned anti-inflammatory mechanisms are mostly derived from invasive VNS, and in a recent systematic review, the results suggest that VNS is only beneficial in acute inflammatory events. The current evidence is insufficient to confirm the claim that VNS affects human inflammatory cytokines (Schiweck et al., 2024). Therefore, further high-quality research is needed to substantiate the anti-inflammatory mechanisms of tVNS in the future.

1. Functional Connectivity and Neuroplasticity

The term “functional connectivity” emerged as a topic term in the keyword cluster with high silhouette values (0.713). tVNS has been demonstrated to optimize brain plasticity, enhancing functional connectivity between brain regions and modulating neurotransmitter and neurotrophic factor secretion (Brambilla-Pisoni et al., 2022). Recent studies have demonstrated that tVNS can enhance functional connectivity between parietal and temporal lobe regions and that it can induce heightened activity in various brain regions among patients with mild cognitive impairment (MCI). Among these patients, the cingulate gyrus may be a particularly promising target for tVNS-based MCI regulation (Chunlei, 2023). The functional impairment of the thalamocortical connectivity network is considered the basis of migraine pathophysiology. The findings of Zhang et al. (2021) confirm that taVNS may alleviate migraines by increasing connectivity between the motor-related subregions of the thalamus and the pregenual anterior cingulate cortex/medial prefrontal cortex while reducing connectivity between the occipital cortex-related thalamic subregion and the central posterior cingulate/precuneus. It is well established that the dynamic equilibrium between excitability and inhibition (E/I balance) is crucial for the development and sustenance of typical cerebral functionality and plasticity (He et al., 2018). VNS has been demonstrated to enhance the imbalance between excitability and inhibition in the brain (Ooi et al., 2023). For instance, previous studies demonstrated that taVNS enhanced the activity of the cortical GABA system and inhibited the release of the excitatory neurotransmitter glutamate (Chen et al., 2016; van Midden et al., 2023). This may be one of the result that VNS can influence the progression of disorders such as epilepsy, stroke, and depression (Di Lazzaro et al., 2004; Bajbouj et al., 2007; Wang et al., 2023). Moreover, research indicates that taVNS may also be an effective treatment for migraines by activating the descending pathways of the locus coeruleus and the dorsal raphe nucleus. These pathways are responsible for the synthesis and release of norepinephrine and serotonin, respectively, which are key neurotransmitters involved in central pain modulation (Hao Han et al., 2023). Baig et al. (2022) demonstrated that prolonged tVNS intervention is associated with increased PPAR-γ, BDNF, and growth differentiation factor 11 in stroke models. These findings suggest that tVNS may promote angiogenesis and neurogenesis, and modulate neuroplasticity, thereby alleviating vascular and neurological impairments linked to stroke.

1. LC-NE system

Keyword cluster #2 “locus coeruleus,” #3 “brain stem” and “norepinephrine” indicate that the mechanism of action of tVNS is related to the LC-NE system. The LC in the brainstem is one of the main sources of NE in the brain. Previous studies have shown that stimulation of the vagus nerve affects the LC-NE system, thereby exerting a therapeutic effect on depression, chronic pain, post-traumatic stress disorder, neurodegenerative diseases, as well as cognitive decline in aging (Kaczmarczyk et al., 2017; Fischer et al., 2018; Zhang et al., 2019; Garcia et al., 2021). Zhang et al. (2019) showed that taVNS at 1 HZ reduced the fMRI signal of LC and enhanced the resting-state functional connectivity between LC and temporoparietal junction, amygdala, hippocampus/parahippocampus, and the left secondary somatosensory cortex, thus modulating the vagal pathway and pain-modulating network to alleviate migraine symptoms in patients with migraine. Additionally, evidence indicates that taVNS may facilitate associative memory and situational memory, while also eliminating fear memory by activating the LC-NE pathway (Hansen, 2018). Furthermore, it has been demonstrated to enhance mood and to promote attention, memory, and cognitive control-factors affected by long COVID (Colzato et al., 2023).

#### 4.2.2 tVNS stimulation patterns (keyword cluster #7)

Keyword cluster #7 (taVNS) is one of the tVNS stimulation patterns. tVNS stimulation modes can be classified into three broad categories: taVNS, transcutaneous cervical vagal nerve stimulation (tcVNS), and transcutaneous vagal nerve magnetic stimulation. These categories are based on the different stimulation sites and modes. Concerning stimulation parameters, it is worth noting that there is no uniform standard. Previous studies have shown that the stimulation frequency is mostly 25 Hz, and the stimulation intensity is often adjusted according to the sensitivity threshold. Pulse widths of 0.2 ms and 0.5 ms are predominant (Yap et al., 2020). However, due to the large variation in stimulation protocols across studies, it may be difficult to determine the optimal stimulation parameters. Therefore, it is suggested that closed-loop controlled tVNS may provide greater clinical benefits.

At present taVNS is widely used as a neuromodulation modality. The most in-depth research is conducted by Prof. Rong Peijing’s team. According to anatomical studies the auricular branch is the only branch of the vagus nerve that reaches the body surface (Butt et al., 2020). taVNS is a promising technique that combines vagus nerve stimulation with Chinese medicine auricular acupoint therapy. The technique aims to regulate bodily functions and treat disease through non-invasive electrical stimulation of skin receptor sites distributed by the auditory branch of the vagus nerve in the outer ear (Wang et al., 2021). According to Jiakai et al. (2023) taVNS and VNS share a common anatomical basis and mechanism of action. The indications and efficacy of VNS and taVNS were also compared and the results indicated that their clinical efficacy was comparable. taVNS has the potential to expand the applications of VNS to include heart failure diabetes mellitus and neurological disorders. However further clinical studies are needed to confirm the efficacy of taVNS.

The research team led by Brock C. is engaged in studies pertaining to tcVNS (Drewes et al., 2021; Kornum et al., 2024). tcVNS is a therapeutic approach that targets the vagus nerve near the carotid artery through transcutaneous electrical stimulation. It is based on the concept of the “carotid bifurcation,” which was initially developed by American neurologist James L. Corning (Farmer et al., 2020). tcVNS was originally developed for the treatment of epilepsy. Its function is to stimulate the branches of the cervical nerve near the carotid artery, which reduces the heart rate (HR) and subsequently decreases blood flow to the brain. As research progressed, tcVNS has gained FDA approval for the management of migraines and cluster headaches (Nesbitt et al., 2015). A multitude of studies have indicated its efficacy in the intervention of cardiovascular diseases, acute ischemic brain disorders, and psychiatric conditions (Ay et al., 2016; Brock et al., 2017; Gazi et al., 2022). In a randomized double-blind controlled trial, Gurel et al. (2020) demonstrated that tcVNS can regulate the autonomic nervous system, cardiovascular, and vascular indices in patients with post-traumatic stress disorder. This treatment reduced sympathetic arousal and improved recovery from traumatic stress. In a study by Moazzami et al. (2023), the levels of gastrin, a biomarker of stress, were measured in response to various stressful stimulation. The results demonstrated that tcVNS can reduce gastrin levels and modulate hormonal and autonomic responses to stress, thereby treating psychiatric disorders.

Transcutaneous vagus nerve magnetic stimulation is an intervention that combines vagus nerve stimulation with repetitive transcranial magnetic stimulation (rTMS; Lin et al., 2018). This innovative approach addresses some limitations of tVNS, including attenuation of current upon entering the body, difficulty in stimulating deep tissues and nerves, and excessively high current intensity, which can lead to adverse events such as pain, skin reddening, and itching (Redgrave et al., 2018). Previous studies have shown that rTMS can activate the auricular branch of the vagus nerve to improve swallowing function in stroke patients (Lin et al., 2018) and patients’ level of consciousness (Wang et al., 2022). Furthermore, Zhang et al. (2023) demonstrated through a single-arm study that transcutaneous cervical vagus nerve magnetic stimulation can effectively enhance cognitive function in patients with traumatic brain injury-related cognitive impairment, offering a safe and feasible treatment option. However, the currently available evidence is insufficient to demonstrate the efficacy and mechanisms of action of transcutaneous vagus nerve magnetic stimulation. Therefore, further clinical and basic research of the highest quality is required to provide more definitive evidence on this topic.

As research on non-invasive neurostimulation techniques progresses, researchers propose the concept of closed-loop transcutaneous auricular vagus nerve stimulation (CL-taVNS), an automated taVNS system regulated by biofeedback signals such as behavioral changes, respiratory variations, and brain activity (Cook et al., 2020). The CL-taVNS system primarily consists of biological signal sensors (identifiers) and taVNS stimulators integrated with remote control solutions (Kaniusas et al., 2019). It aims to adapt more sensitively to dynamically detectable changes in the clinical setting, thus providing personalized taVNS protocols to enhance therapeutic efficacy. Current forms of CL-taVNS include movement-activated auricular vagus nerve stimulation (MAAVNS) and respiratory-gated auricular vagus nerve afferent stimulation (RAVANS). MAAVNS has been applied in neonatal neurorehabilitation (Badran et al., 2020) and adult upper limb rehabilitation. RAVANS have been previously utilized in intervention studies for the treatment of pain (Garcia et al., 2017) and hypertension (Stowell et al., 2019). Moreover, in the future, electroencephalogram signals, electrocardiogram signals, and subcutaneous fluid signals may also be considered as potential triggers for taVNS in specific patients (Yu et al., 2021). Nevertheless, given the limited sample sizes and the paucity of research currently available, the efficacy and safety of these approaches remain uncertain. It is conceivable that this will become a prospective avenue of development for non-invasive neuromodulation techniques in the future.

#### 4.2.3 tVNS advantageous diseases (keyword clusters #1 and #5)

##### 4.2.3.1 Diseases of the nervous system

Keyword clusters #1 and #5 showed the parts of tVNS advantageous diseases. Currently, there is significant attention being given to tVNS intervention in neurological disorders, including migraines, Parkinson’s disease, Alzheimer’s disease, epilepsy, and stroke. Prof. Liebler Eric’s team delves into the efficacy of tVNS intervention in cluster headaches and related mechanisms (Diener et al., 2019). The findings of their study indicated that tVNS was an efficacious intervention for the acute and prophylactic treatment of migraine headaches (Grazzi et al., 2017; Goadsby et al., 2018). According to a meta-analysis, tVNS has significantly increased the responder rate by at least 50% in migraine patients. Low-frequency taVNS has been found to reduce the number of migraine days significantly. Furthermore tcVNS is considered safe and well-tolerated (Song et al., 2023). A four-week clinical trial conducted by Zhang et al. (2021) demonstrated that taVNS could relieve headache symptoms and modulate thalamocortical circuits in migraine patients. These findings suggest a potential therapeutic target for this population. Research has shown that tVNS affects the cortical areas responsible for controlling trigeminal pain. Additionally, taVNS (1 Hz) significantly alters the activity and connectivity of the central vagal pathway and brain regions associated with the pain modulatory system. These findings may provide insight into the neural mechanisms of taVNS for treating migraines (Zhang et al., 2019).

In our analysis the terms “memory” and “Alzheimer’s disease” emerged as key concepts with high frequency. AD is a neurodegenerative disease commonly observed in the elderly population. It is characterized by the formation of neuroinflammatory plaques made up of amyloid β-protein (Aβ) and neurofibrillary tangles made up of hyperphosphorylated tau proteins. AD is characterized by atrophy in various regions of the brain including the hippocampus and internal olfactory cortex. Neuromodulation techniques are being investigated as a potential treatment for AD (Duyu and Wei, 2019). One such technique is tVNS which has demonstrated encouraging outcomes in animal studies. In particular tVNS has been shown to alter the morphology of microglia in aged AD model animals such as APP/PS1 mice from a neurodestructive phenotype to a neuroprotective phenotype. This has the potential to slow down the progression of the disease (Kaczmarczyk et al., 2017). Recent studies have shown that 40 Hz taVNS may inhibit hippocampal P2X7R/NLRP3/Caspase-1 signaling and potentially improve spatial learning and memory in APP/PS1 mice (Yu et al., 2023). However further clinical research is needed to determine the effectiveness of tVNS in treating AD.

The keyword co-occurrence analysis revealed that the term “Parkinson’s disease” appeared 18 times, which suggests that tVNS may be an effective therapy for Parkinson’s disease (PD). Previous evidence suggests that tVNS may have beneficial therapeutic effects on both motor and non-motor symptoms in PD. It has been proposed that tVNS could potentially improve several objective gait parameters, such as stride length, speed, and frequency (Marano et al., 2022). In a study conducted by Zhang et al. (2023), the effects of tVNS (20 Hz) on gait disturbances in PD patients were investigated. The results demonstrated that tVNS was an effective intervention for alleviating gait disturbances and remodeling sensorimotor integration. Lench et al. (2023) also conducted a study that affirmed the safety and feasibility of multiple sessions of taVNS in intervening PD. Furthermore, the involvement of the vagus nerve in regulating the onset and progression of PD has prompted researchers to propose tVNS as a potential treatment for autonomic dysfunction in PD (Ko, 2021).

The keyword “epilepsy” is referenced with greater frequency. Among the 10 most frequently cited documents are studies on tVNS for epilepsy (Stefan et al., 2012). VNS has previously been used to manage refractory epilepsy (Penry and Dean, 1990), but due to safety and tolerability concerns associated with implantable VNS, tVNS has emerged as a potential alternative therapy (Ben-Menachem et al., 2015). In a large randomized controlled study, Yang et al. (2023) investigated the effectiveness and safety of tVNS in patients with epilepsy. The study showed a significant reduction in seizure frequency after the intervention, and no serious adverse events were observed. These findings suggest that tVNS may be a safe and effective treatment option for epilepsy. In a study conducted by von Wrede et al. (2022), the effects of taVNS on brain network function in various types of epilepsy were investigated. The study found that taVNS produced immediate and significant improvements in network robustness. However, it is worth noting that the lasting effects of taVNS differed significantly across types of epilepsy. While the focal epilepsy group experienced enhanced robustness, the generalized epilepsy group experienced a reduction in robustness. The fluctuating stability of the network could potentially be linked to the magnitude of the susceptibility to perturbation induced by taVNS in different types of epilepsy.

The results of the top 25 Keywords with the Strongest Citation Bursts indicate that “stroke” is a more popular research topic in the period from 2022 to 2024. The team led by Schaller demonstrated that tVNS can enhance limb and memory function in patients with ischemic stroke by increasing central noradrenergic activity (Sternberg and Schaller, 2020). Furthermore, tVNS has been demonstrated to markedly enhance upper limb motor function, cognitive, and dysphagia in stroke patients (Yuan et al., 2019; Colombo et al., 2023). The mechanism of action may be related to various factors, some acute effects including the reduction of infarct size, improvement of neurological deficits, regulation of blood–brain barrier permeability and inhibition of neuroinflammation. Longer term effects of tVNS in stroke that may mediate neuroplasticity include microglial polarization, angiogenesis and neurogenesis (Baig et al., 2022, 2023). It has been confirmed that tVNS intervention is both effective and safe in treating nervous system diseases. However, the mechanism of action is still not fully understood and requires further exploration in the future.

##### 4.2.3.2 Mental illness

The research team led by Prof. Peijing Rong has dedicated the past few years to investigating the efficacy and mechanism of tVNS in treating depression (Sun et al., 2024). In 1997 and 2005, respectively, the FDA approved the use of cervical VNS for refractory depression that does not respond to pharmacological treatments (Cristancho et al., 2011). In 2016, Rong et al. (2016) conducted a non-randomized controlled pilot study to explore the effects of taVNS on MDD. After 12 weeks of intervention, it was observed that the taVNS group showed a more significant improvement in their symptoms compared to the control group after 4 weeks of treatment. This improvement was mainly observed in terms of changes in Hamilton scores, as well as response and remission rates in the fourth week. A recent study further investigated the effects of prolonged longitudinal taVNS on the modulation of functional connectivity in striatal subregions of MDD patients. According to the study, prolonged longitudinal taVNS was found to affect the resting-state functional connectivity in the striatum with the prefrontal cortex, occipital cortex, and temporal cortex. It was also found that this effect was associated with symptom improvement (Zhang et al., 2022). Furthermore, Wang et al. (2021) conducted an experimental study utilizing CUMS rats as a model, which demonstrated that taVNS may exert antidepressant effects by modulating the hippocampal α7nAchR/NF-κB signaling pathway.

##### 4.2.3.3 Diseases of the circulatory system

The second most frequent keyword is “heart rate variability,” which indicates that tVNS has been met with considerable enthusiasm in the field of cardiovascular disease. Current studies have shown that tVNS is effective in improving heart rate variability and maintaining the balance of the autonomic nervous system (Geng et al., 2022). In a study conducted by Clancy et al. (2014), the effects of tVNS on autonomic function were investigated in 48 healthy participants. The results demonstrated that tVNS enhanced heart rate variability and reduced sympathetic outflow, which has significant implications for the clinical management of disorders with elevated sympathetic activity, such as heart failure. Central blood pressure is considered to be the main indicator of left ventricular (LV) afterload, so lowering central blood pressure can reduce LV afterload and prevent heart failure decompensation. One study showed that tVNS in the left external auditory canal can significantly reduce central blood pressure in elderly patients with acute heart failure (AHF), reduce cardiac afterload, and thus improve cardiac function in patients with AHF (Nagai et al., 2023).

tVNS has also been shown to be effective in arrhythmias and myocardial infarction. Previously, it has been suggested that low levels of tVNS may exert anti-fibrillation effects by prolonging the effective refractory period of atrial and pulmonary vein myocardium, inhibiting activation of the atrioventricular ganglionic plexus, decreasing stellate ganglionic neural activity, and decreasing sympathetic ganglion cells in the sympathetic left stellate ganglion (Li et al., 2015). Dalgleish et al. (2021) have further demonstrated that occipital artery decompression in combination with taVNS increases cardiac parasympathetic tone on the one hand and prolongs atrial conduction time on the other hand, which has a positive effect on ventricular rate control during auricular fibrillation. There is also evidence that taVNS may ameliorate cardiac ischemia/reperfusion injury by mediating the dynamic balance between pro-inflammatory and anti-inflammatory responses in cardiac macrophages (Chung et al., 2020). This suggests that tVNS may play a beneficial role in improving cardiac function and regulating cardiac rhythm.

##### 4.2.3.4 Diseases of the digestive system

Citation clusters #4 (postoperative ileus) and #8 (electrogastrogram) appear with greater frequency. It is evident that aside from circulatory system and neuropsychiatric diseases, tVNS also impacts the development of digestive system diseases. Studies indicate tVNS may be an effective treatment for gastrointestinal discomfort in PD (Kaut et al., 2019). Steidel et al. (2021) demonstrated tVNS’s ability to enhance gastric motility in healthy individuals, particularly with high-frequency stimulation. Furthermore, tVNS has demonstrated efficacy in alleviating constipation and abdominal discomfort in patients with irritable bowel syndrome. This effect may be mediated by autoimmune mechanisms such as activation of the CPA, inhibition of the 5-HT pathway, and improvement of rectal sensation (Shi et al., 2021). Additionally, tVNS has been observed to have a combined effect on visceral hypersensitivity, delayed gastric emptying, and depression-like behavior in iodoacetamide (IA)-treated rats. These effects are likely linked to anti-inflammatory activation, improvement of duodenal mucosal integrity, enhanced vagal efferent activity, and down-regulation of HPA axis hyperactivation (Hou et al., 2023). Moreover, Müller et al. (2022) demonstrated that tVNS enhanced gastro-brain coupling via the NTS-midbrain pathway, thereby substantiating the capacity of vagal signaling to effectively modulate brain-digestive organ communication and the beneficial impact of vagal modulation in digestive disorders.

#### 4.2.4 tVNS safety

While tVNS is widely recognized as a promising replacement therapy for VNS in clinical practice due to its non-invasiveness and effectiveness, researchers remain concerned about its safety. The results of the keyword co-occurrence analysis also showed that keyword “safety” appeared frequently. The underlying cause of its safety concerns may be linked to the TCR (Chowdhury et al., 2014). Previous studies has demonstrated that when the trigeminal nerve is stimulated, excitation is conveyed to the vagus nerve via the common fiber ganglion, resulting in an enhancement of vagal excitability and subsequent cardiovascular effects, including an increase in heart rate and blood pressure (Meuwly et al., 2017). It is generally believed that tVNS is mostly applied to the left ear, as stimulation of the right side is more likely to induce bradycardia since the efferent vagal fibers on the right side regulate heart rate (Keute et al., 2018). It is worth noting that tcVNS tends to stimulate peripheral non-vagal nerves. Similar to invasive VNS, it can be challenging for tcVNS to selectively stimulate VN fibers percutaneously. Consequently, current products are likely to stimulate afferent and efferent fibers indiscriminately. Some have questioned the safety of this modality due to the possibility of stimulating motor efferent nerves that innervate the sinus node, which could lead to arrhythmias, conduction block, and other adverse effects (AEs; Simon and Blake, 2017). However, analyzing relevant clinical studies in recent years, the incidence of AEs associated with tVNS was low. A recent meta-analysis evaluated the possible AEs of taVNS and their incidence. The study found that the most common AEs reported were earache, headache, tingling, dizziness, skin redness, and fatigue. However, the results showed no significant difference in the risk or intensity of AEs between the taVNS group and the control group, suggesting that taVNS is a safe treatment option (Kim et al., 2022). It has been shown that tVNS can produce therapeutic effects similar to those of VNS but with a higher level of safety. Additionally, tVNS is effective in treating refractory epilepsy, particularly in pediatric cases (Rong et al., 2014). Clinical studies have evaluated the efficacy and safety of tVNS in treating acute stroke. The studies have shown that both tcVNS and taVNS are safe and effective in treating acute ischemic or hemorrhagic stroke (Arsava et al., 2022; Li et al., 2022). Additionally, tVNS is safe in treating acute/chronic headaches, depression, gastrointestinal disorders, and Novel coronavirus pneumonia (Goadsby et al., 2018; Kaczmarczyk et al., 2021; Tornero et al., 2022; Alam and Chen, 2023). It is suggested that tVNS may be comparatively safer than VNS. However, it is important to note that the current studies have mostly included small sample sizes and short follow-up times. Therefore, it is recommended that more large clinical studies be conducted in the future to validate the safety of tVNS further.

## 5 Conclusion and outlook

This bibliometric study reveals the global publication trends and dynamics of tVNS in the 21st century.The results show that the research fever in the field of tVNS is generally on the rise. The study systematically clarifies the development of this research field and comprehensively analyzes the current hot research topics related to tVNS, including mechanism of action, stimulation mode, advantage diseases, and safety. This will help to grasp the future development direction of tVNS.

The current tVNS research field still faces some challenges. Firstly, the mechanism of tVNS mainly focuses on exploring the anti-inflammatory pathway, and its central and peripheral anti-inflammatory effects have been verified by some clinical and basic experiments, but the relevant research is still insufficient. There is a relative lack of research on the application and mechanism of tVNS in acute and chronic inflammation of various respiratory and digestive diseases.

Secondly, It is well known that VN has been confirmed as a crucial mediator of bidirectional communication between the gut and the brain (Bonaz et al., 2018). Consequently, the brain-gut axis may also be a mechanism of action of tVNS. Although there are fewer related studies, this may be one of the future research directions.

Thirdly, the current research is somewhat arbitrary in its selection of tVNS treatment sites and stimulation parameters. Previous studies indicated that different diseases may respond differently to specific stimulation parameters. The use of tVNS for the treatment of epilepsy and depression involves the application of a range of stimulation frequencies (20–30 Hz), pulse widths (up to 500 μs), and stimulation on-times (30–90 s) followed by off-times (5 min). The optimal parameters for the modulation of cognition require the use of much higher current stimulations, typically up to 8 mA (Vargas-Caballero et al., 2022). In contrast, Yu et al. (2023) demonstrated that 40-Hz taVNS enhanced spatial learning and memory in APP/PS1 mice. The findings of Badran et al. (2019) indicate that the 500 μs pulse width is the most biologically active. In terms of frequency, 25 Hz has proven to be an effective frequency. Nevertheless, further studies are required in the future in order to establish standardized tVNS stimulation protocols for different diseases.

Finally, there is still a lack of evidence regarding the longer-term efficacy and safety of tVNS, In the future, more clinical studies with large samples and long-term follow-up are needed to validate.

It is predicted that tVNS research will continue to focus on the effects of tVNS on neuropsychiatric diseases in the next few years and explore its mechanism of action in depth. It is also expected to strengthen the combination with ECG, EEG, and other technical means to form a closed-loop automated stimulation, to take advantage of its precise neuromodulation. In addition, the combination of tVNS with other non-invasive neuromodulation techniques (e.g., TMS) may be a potential direction for future research.

This study is subject to certain limitations. Firstly, only the WOS database was used as the source of literature analysis. Secondly, as there was less tVNS-related literature before 2000, only literature published after the 21st century was included in this study. This may result in some errors and biases. Nevertheless, an analysis of global publications since the 21st century allows for the visualization of publishing trends and promising areas of research in the dynamic development of tVNS. This analysis informs the establishment of new research directions for tVNS.
