# Supplementary material for: Transcutaneous vagus nerve stimulation: a bibliometric study on current research hotspots and status
Source: Front Neurosci. 2024 Aug 16;18:1406135. doi: 10.3389/fnins.2024.1406135 (PMC11363710; doi:10.3389/fnins.2024.1406135)
Supplement: Supplementary file 1 [file Data_Sheet_1.PDF]

Supplementary Material

1 Supplementary Figures and Tables

Supplementary Table 1 The Top 10 Authors in tVNS Research

English Search Strategies (Web of Science)
Topic=Transauricular Vagal Nerve Stimulation OR Transauricular Vagus Nerve Stimulation OR Transcutaneous Auricular Vagus Nerve Stimulation OR taVNS OR tVNS OR Transcutaneous Auricular Vagal Nerve Stimulation OR Non-Invasive vagus nerve stimulation OR Non-Invasive vagal nerve stimulation OR Transcutaneous Auricular Vagus Nerve OR Transcutaneous Auricular Vagal Nerve OR Transcutaneous cervical vagus nerve stimulation OR tcVNS OR Transcutaneous cervical vagal nerve stimulation OR Transcutaneous cervical vagal nerve OR Transcutaneous cervical vagus nerve OR Non-Invasive vagal nerve OR Non-Invasive vagus nerve OR Transauricular Vagal Nerve OR Transauricular Vagus Nerve OR taVN OR tVN OR tcVN

Supplementary Table 2 The Top 10 Authors in tVNS Research

Rank	Number of publications /article	Institute	Author
1	52	Institute of Acupuncture and Moxibustion, China Academy of Chinese Medical Sciences	Peijing,Rong

2	24	Guang'anmen Hospital, China Academy of Chinese Medical Sciences	Jiliang,Fan g
3	22	Institute of Acupuncture and Moxibustion, China Academy of Chinese Medical Sciences	Yu,Wang
4	21	Institute of Acupuncture and Moxibustion, China Academy of Chinese Medical Sciences	Shaoyuan, Li
5	16	Department of Psychiatry, Massachusetts General Hospital, Harvard Medical School	Kong, Jian
5	16	Institute of Acupuncture and Moxibustion, China Academy of Chinese Medical Sciences	Zhang, Yue
6	14	ElectroCore, Inc., Basking Ridge	Liebler, Eric
6	14	Departments of Psychiatry & Behavioral Sciences, Emory University School of Medicine	Douglas Bremner J
6	14	Department of Psychiatry and Behavioral Sciences, Medical University of South Carolina	Badran, Bashar W
10	13	Department of Epidemiology Rollins School of Public Health Emory University	Amit J. Shah
